# An Investigation of the Effectiveness of the Family Adoption Program Among Undergraduate Medical Students

**DOI:** 10.7759/cureus.92582

**Published:** 2025-09-17

**Authors:** Panchasheela S Reshmi, Geeta K Patel, Harsh K Patel, Paresh R Prajapati, Ruchita T Lunagariya, Kinnari I Gupta, Malay P Savalia

**Affiliations:** 1 Department of Community Medicine, SAL Institute of Medical Sciences, Ahmedabad, IND

**Keywords:** affective, cognitive, family adoption program, holistic, psychomotor

## Abstract

Background

The National Medical Commission introduced the Family Adoption Program (FAP) under Competency-Based Medical Education to promote holistic learning across cognitive, psychomotor, and affective domains. Rooted in the Sewagram model, FAP emphasizes experiential, community-based learning to improve medical students’ communication skills, clinical competence, and understanding of rural health. This initiative aims to bridge the urban-rural healthcare gap while nurturing empathetic and socially accountable healthcare professionals. In this study, we aimed to assess the effectiveness of FAP in enhancing cognitive, psychomotor, and affective domains among medical students.

Methodology

A prospective, longitudinal study was conducted among 150 MBBS students over 14 months. Students’ performance in the three domains was evaluated before and after participation using a validated semi-structured questionnaire. Paired t-tests were used to analyze cognitive and psychomotor scores, while affective responses were summarized descriptively.

Results

A significant improvement was observed in both knowledge and psychomotor skills after FAP participation. Mean cognitive scores increased from 22.5 ± 6.5 to 31.6 ± 5.6, and psychomotor scores increased from 16.6 ± 7.2 to 29.0 ± 6.3 (p < 0.001). Post-test results indicated better understanding and practical abilities. In the affective domain, all students found FAP useful, and 90.7% reported enhanced community knowledge. Most students highlighted the importance of ethical values, teamwork, and accountability, recognizing FAP as a meaningful experiential learning initiative.

Conclusions

FAP significantly enhanced cognitive and psychomotor skills, while the affective domain revealed improved rapport with families, greater community insight, and strengthened values of ethics, teamwork, and social responsibility.

## Introduction

The National Medical Commission (NMC) introduced Competency-Based Medical Education (CBME) for MBBS courses starting in August 2019. This curriculum, overseen by the Undergraduate Medical Education Board, emphasizes a holistic approach to medical training by covering all three domains of learning, namely, cognitive, affective, and psychomotor [1]. CBME marks a paradigm shift in medical education, focusing on the acquisition of essential skills and abilities necessary for effective patient care [2].

One of the significant components of CBME is the inclusion of the Family Adoption Program (FAP), introduced through NMC’s notification dated March 31, 2022. This initiative aligns with the broader objective of instilling humanistic qualities and understanding the social determinants of health in medical graduates [3]. FAP is conceptualized on the Sewagram model developed by the Mahatma Gandhi Institute of Medical Sciences, Sewagram [4]. It aims to provide an experiential learning opportunity to Indian medical graduates toward community-based healthcare and thereby enhance equity in health. This program caters to young students to improve their communication skills, learn to analyze data, understand rural dynamics, identify diseases, and come up with ways to improve the standards of rural families [5].

Under FAP, every medical student is mandated to adopt a minimum of three families, ideally five [6]. The program is executed under the guidance of faculty from the Department of Community Medicine [1]. Students monitor the general health of these families, provide health-related advice, assist them in accessing healthcare facilities, and follow up on their health progress. Notably, villages selected for FAP must not overlap with areas already covered under the Rural Health and Training Centre [7].

The need for FAP arises from the stark contrast between the geographical distribution of healthcare facilities and population density in India. Approximately 65% of the population resides in rural areas, while healthcare facilities are predominantly concentrated in urban regions. This disparity, compounded by a lack of health literacy and awareness, contributes to poor healthcare-seeking behavior and adverse health outcomes in rural communities [8]. FAP addresses these challenges by serving two primary purposes, i.e., improving rural residents’ access to healthcare and equipping future healthcare practitioners with community-focused training [7].

Several studies have highlighted the benefits of “community as a classroom” in medical education. This approach fosters the development of communication skills, empathy, leadership, and an understanding of cultural and social factors affecting health. It also enables students to act as primary consultants for households, hone diagnostic and management skills, and gain training in family medicine [9-12]. The NMC has initiated the FAP in an attempt to produce medical graduates with a community health perspective, thereby ensuring that the services of medical professionals are accessible to all citizens. This would, in turn, facilitate the achievement of the national health goals [13].

The FAP was introduced as an innovative approach in undergraduate medical education to foster a holistic learning experience encompassing cognitive, affective, and psychomotor domains. By engaging students directly with families, the program aims to enhance their clinical knowledge, understanding of social determinants, rural dynamics, and community-based healthcare, which are vital for empathetic and patient-centered medical practice. Assessing the effectiveness of FAP is crucial to determine its impact on students’ development across these domains and identify areas for improvement. Such evaluation provides evidence for its value in shaping competent and compassionate healthcare professionals. Furthermore, it supports the refinement of medical education strategies, ensuring alignment with modern healthcare needs and standards set by the Undergraduate Medical Education Board.

This study evaluates the effectiveness of the FAP in enhancing medical students’ cognitive, psychomotor, and affective domains under the CBME framework. Given the rural-urban healthcare disparity in India, FAP aims to bridge this gap by promoting community-based learning.

## Materials and methods

Study design and participants

This prospective, longitudinal study was conducted among MBBS students over a period of 14 months, from January 2024 to February 2025. A total of 150 participants were selected using purposive sampling. In the first year of the MBBS, students who had not received any orientation on the FAP before its commencement were classified as pre-test participants. After completing nine FAP visits during Phase I and ten visits during Phase II of their MBBS curriculum, they were classified as post-test participants.

Inclusion and exclusion criteria

The study included first-year MBBS students enrolled in the FAP during the study period who had not previously participated in similar community-based programs and who provided informed consent to participate. Students were excluded if they were absent for more than two FAP visits, declined to participate, or submitted incomplete questionnaires.

Study tool

The study tool (Appendices) consisted of a semi-structured questionnaire developed by the authors following an extensive literature review and consultation with experts. Content and face validity were ensured through review by faculty members experienced in community-based medical education. The questionnaire addressed the three domains. The cognitive domain, which included 42 multiple-choice and short-answer questions divided into four sections, i.e., Family Structure and Dynamics (10 questions), Environment (17 questions), Socioeconomic Status and Demographics (9 questions), and Health Screening and Measurements (6 questions). The psychomotor domain, which included 39 items assessing skills across five sections, i.e., Blood Pressure Measurement (4 questions), Mid-Upper Arm Circumference (MUAC) Measurement (6 questions), Infant Length and Height Measurement (13 questions), Weight Measurement (6 questions), and Random Blood Sugar (RBS) Measurement (10 questions). The affective domain, which included 12 descriptive and Likert-scale items designed to explore ethical values, teamwork, accountability, social commitment, and responsibility.

Data collection

Data were collected using Google Forms, and the link was shared with the students. Participants were required to complete the form, ensuring anonymity and confidentiality.

Data analysis

The data were analyzed after ensuring completeness. Scoring was applied to the cognitive and psychomotor domains, with correct responses assigned a score of 1 and incorrect responses assigned a score of 0. The maximum scores were 42 for the cognitive domain and 39 for the psychomotor domain. Statistical analysis of cognitive and psychomotor domain scores was conducted using a paired t-test, with a p-value <0.05 considered statistically significant. Descriptive statistics were used to summarize responses in the affective domain.

## Results

**Figure 1:**
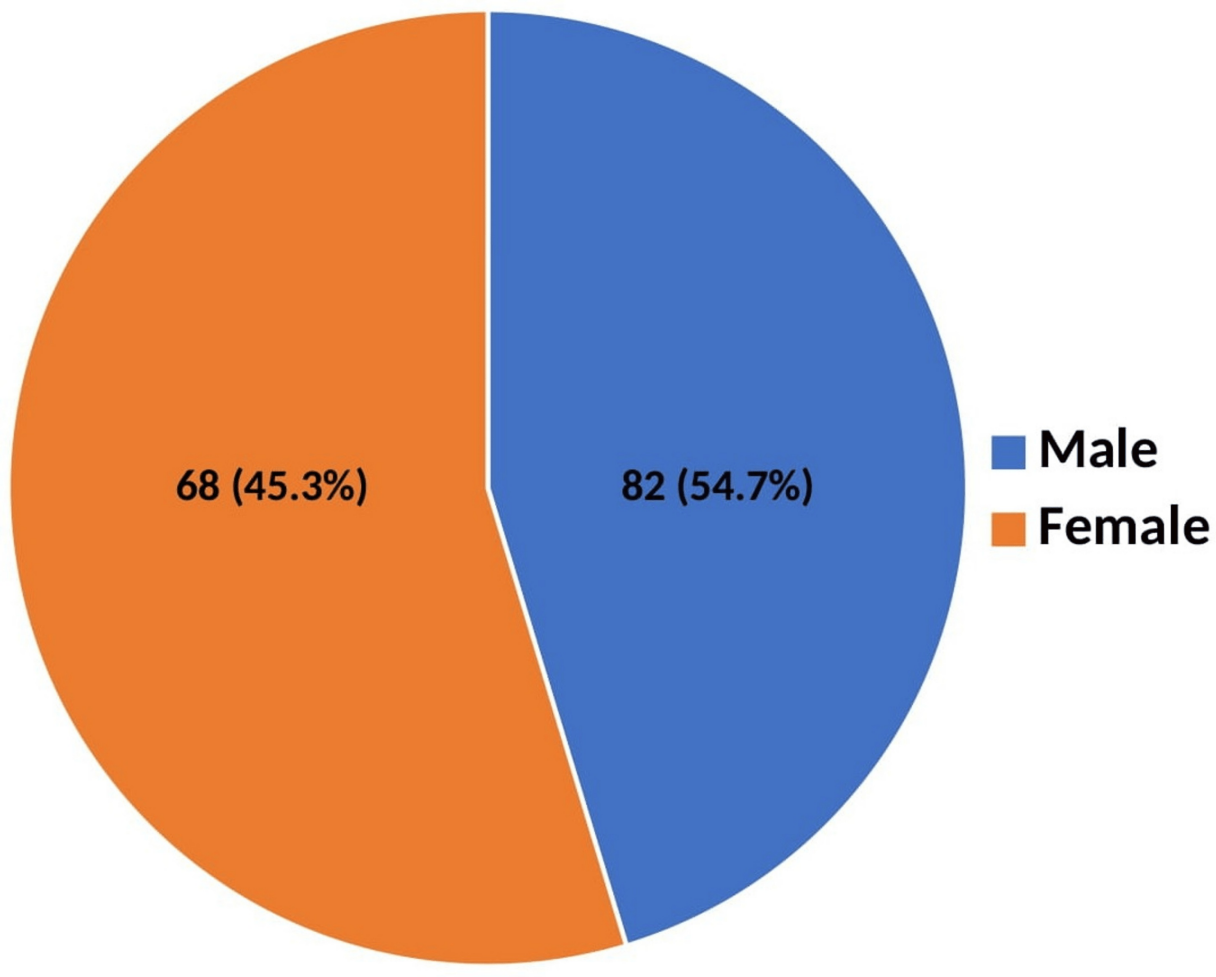
Gender-wise distribution of the study participants (n = 150).

In this study, Batch 2023 MBBS students were chosen as the study participants. The study provided a comparative assessment of the impact of the implementation of FAP. Figure 1 depicts the gender-wise distribution of study participants, with 68 (45.3%) males and 82 (54.7%) females.

Table 1 presents the mean knowledge scores across four sections, i.e., Family Structure and Dynamics (10 questions), Environment (17 questions), Socioeconomic Status and Demographics (9 questions), and Health Screening and Measurements (6 questions). Post-test participants demonstrated higher mean scores across all sections, indicating an enhanced understanding in these areas.

**Table 1:** Mean knowledge scores of pre-test and post-test participants.

Variables	Number of questions	Pre-test		Post-test	
		Mean	SD	Mean	SD
Family Structure and Dynamics	10	5	1.9	7.1	1.8
Environment	17	10.3	2.9	13.3	2.1
Socioeconomic Status and Demographics	9	4.1	2.1	6.6	1.9
Health Screening and Measurements	6	3.1	1.2	4.6	1.2

**Figure 2:**
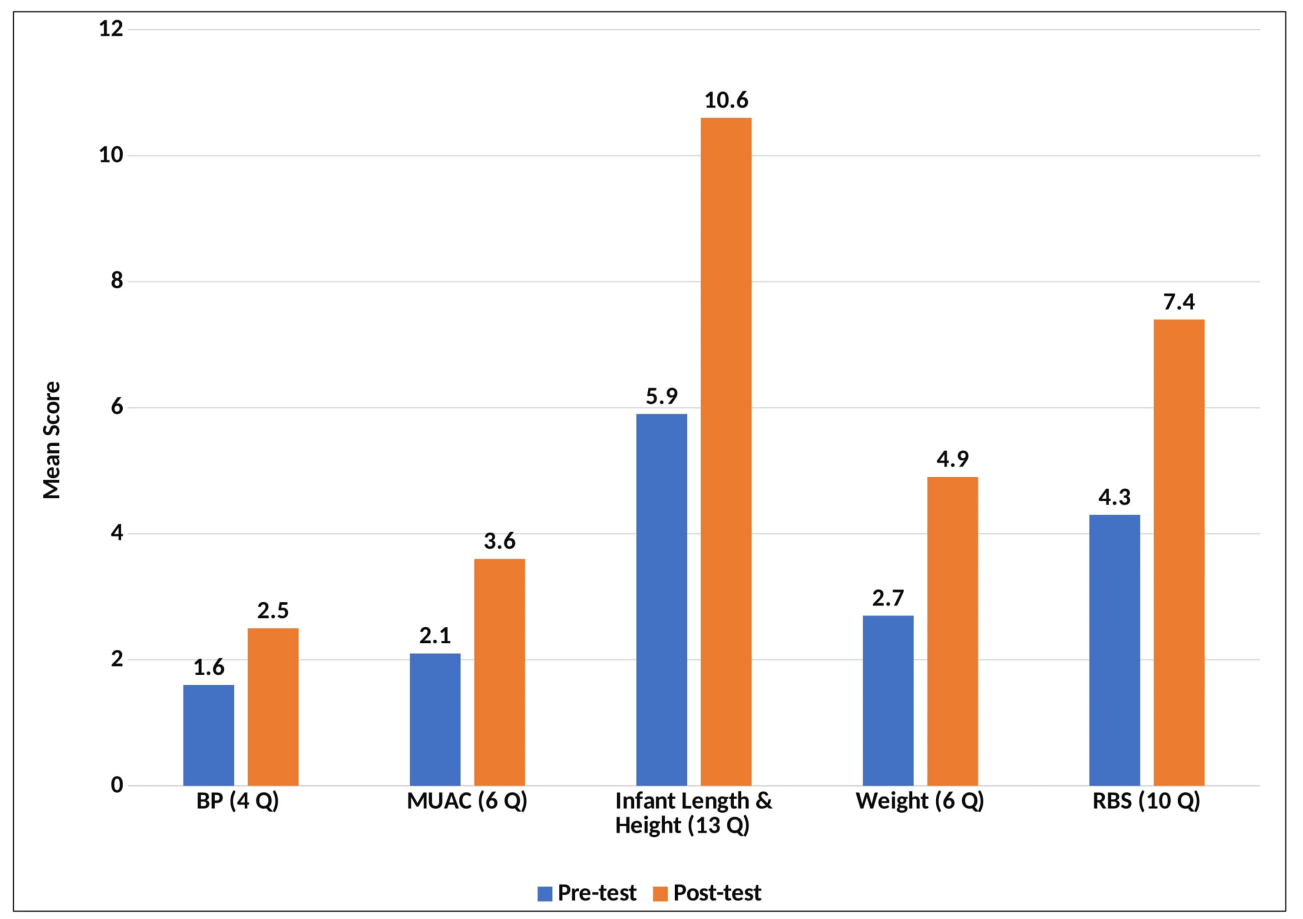
Mean psychomotor skill scores in pre and post-test participants. BP = blood pressure; MUAC = mid-upper arm circumference; RBS = random blood sugar

Figure 2 highlights the mean psychomotor skills scores of participants across five sections, i.e., Blood Pressure Measurement (4 questions), MUAC Measurement (6 questions), Infant Length and Height Measurement (13 questions), Weight Measurement (6 questions), and RBS Measurement (10 questions). Post-test participants demonstrated higher mean scores across all sections, indicating an enhanced understanding in these areas.

Table 2 presents a comparative analysis of the mean scores for knowledge and psychomotor skills before and after the implementation of the FAP. The mean knowledge score increased from 22.5 ± 6.5 pre-FAP to 31.6 ± 5.6 post-FAP, with a statistically significant difference (t = -14.1, p < 0.001). Similarly, the mean psychomotor skills score improved from 16.6 ± 7.2 pre-FAP to 29.0 ± 6.3 post-FAP (t = -16.8, p < 0.001). These findings demonstrate a significant enhancement in both knowledge and psychomotor skills among participants following the implementation of FAP.

**Table 2:** Impact of FAP implementation on knowledge and psychomotor skills: a comparative mean score analysis. *: Statistical test: paired t-test. FAP = Family Adoption Program

Variables		Mean	SD	t-value	P-value
Knowledge score	Pre-test	22.5	6.5	-14.1	<0.001
	Post-test	31.6	5.6		
Psychomotor skills score	Pre-test	16.6	7.2	-16.8	<0.001
	Post-test	29	6.3		

Table 3 summarizes the distribution of participants based on their affective domain related to FAP. The majority of the participants, 103 (68.7%), demonstrated good rapport with adopted families, and all participants, 150 (100%), considered FAP to be useful. Support for solving family problems was primarily provided through counselling, 89 (59.3%), health talks, 72 (48%), and motivation, 49 (32.7%). Most participants, 136 (90.7%), believed FAP enhanced community knowledge, and 103 (68.7%) viewed the process as useful for learning, though 32 (21.3%) described it as challenging but rewarding for field exposure. Ethical values, teamwork, social commitment, and accountability were rated as critical factors by most participants, emphasizing their importance in ensuring the success of FAP.

**Table 3:** Distribution of the study participants according to their affective domain (N = 150). *: Responses were collected using a multiple-choice questionnaire. FAP = Family Adoption Program

Variables	Category	f	%
Rapport with the adopted family	Poor	22	14.7
	Good	103	68.7
	Excellent	25	16.6
Opinion on FAP	Useful	150	100
	Not useful	0	0
Support for solving family problems*	Counselling	89	59.3
	Motivating	49	32.7
	Referral	29	19.3
	Health talk	72	48
Response to uncooperative families	Try to convince	138	92
	Leave that family	12	8
Attitude toward FAP*	Adoption enhances community knowledge	136	90.7
	It’s a waste of time	3	2
	Uncertain of its impact on my study	16	10.7
View on the process of FAP*	Difficult and risky	7	4.7
	Useful for learning	103	68.7
	Challenging but rewarding for field exposure	32	21.3
	Seems too complicated	4	2.7
	Helpful for rural populations	72	48
	Only considered if absolutely necessary	4	2.7
View on FAP challenges*	Prepared to face challenges with patience	97	64.7
	Challenges worry me, but they can be managed	59	39.3
	Challenges might overwhelm me	7	4.7
	I can’t handle the challenges of FAP	4	2.7
Importance of ethical values in FAP	Most important	84	56
	Moderately important	62	41.3
	Not important	4	2.7
View on teamwork in FAP	Most important	98	65.3
	Moderately important	51	34
	Not important	1	0.7
Importance of accountability in FAP success	Most important	71	47.3
	Moderately important	79	52.7
	Not important	0	0
Importance of social commitment in FAP	Most important	101	67.3
	Moderately important	41	27.3
	Not important	8	5.3
Importance of responsibility in FAP	Most important	70	46.7
	Moderately important	76	50.7
	Not important	4	2.7

## Discussion

The present study evaluated the effectiveness of the FAP on cognitive, psychomotor skills, and affective domains among MBBS students. In alignment with these objectives, the study findings revealed a significant improvement in knowledge and psychomotor skills scores in post-test assessments following nine FAP visits during Phase I and ten visits during Phase II of the MBBS curriculum, compared to pre-test scores, which were assessed before any orientation on FAP. These findings are consistent with the results reported by Vairavasolai et al. [14], which indicate that sustained participation in the program significantly enhances learning outcomes.

Our study findings indicate a significant improvement in participants’ knowledge across four key sections following the intervention. The Family Structure and Dynamics section showed an increase from 5.0 (pre-test) to 7.1 (post-test), while the Environment section improved from 10.3 to 13.3. Similarly, the Socioeconomic Status and Demographics section showed improvement from 4.1 to 6.6, and the Health Screening and Measurements section increased from 3.1 to 4.6. These results align with findings from various studies on community-based learning programs, particularly the FAP. Kolb’s experiential learning theory emphasizes that hands-on, community-based learning fosters better knowledge retention and skill acquisition, which supports the positive impact observed in our study [15]. This suggests that active engagement in community settings enhances learning outcomes through real-world applications. A study by Baruah et al. [16] highlighted that FAP contributed to improved knowledge, psychomotor skills, and communication abilities among participants, with 100% of faculty members and over 50% of students acknowledging these benefits. Additionally, the program facilitated a better understanding of social structures and community health status, findings that closely align with our observed improvements in knowledge scores. Similarly, a study by Ganganahalli et al. [17] reported that 90.8% of participants agreed that FAP improved their understanding of patient-family dynamics, while 74.4% felt it positively influenced their academic performance. This supports the notion that structured community-based interventions enhance learning, complementing our study’s evidence of improved knowledge across multiple sections. Shikha et al. [7] emphasized that community-academic partnerships provide a unique opportunity to explore social factors influencing health, a perspective that is evident in the increased post-test scores in our study. The exposure to real-life scenarios likely contributed to participants’ deeper understanding of social and health-related aspects. Furthermore, qualitative insights from Reshmi et al. [18] reinforce these findings, with faculty members noting that students gained knowledge on environmental aspects, vector-borne diseases, and sociodemographics through FAP. This observation is consistent with our study’s significant improvement in the Environment (10.3 to 13.3) and Socioeconomic Status and Demographics (4.1 to 6.6) sections, demonstrating the effectiveness of immersive learning experiences. Overall, our findings are consistent with existing literature, emphasizing that structured, community-based learning interventions such as FAP enhance knowledge acquisition across multiple domains. The improvements observed in our study reinforce the importance of experiential learning in medical education, contributing to both academic and practical competencies.

The psychomotor skill assessment covered five key sections, each with a specific number of questions, showing overall improvement in participants’ proficiency. The Blood Pressure Measurement section (4 questions) saw scores increase from 1.6 (pre-test) to 2.5 (post-test), while the MUAC Measurement section (6 questions) improved from 2.1 to 3.6. The Infant Length and Height Measurement section (13 questions) showed an increase from 5.9 to 10.6. Similarly, the Weight Measurement section (6 questions) improved from 2.7 to 4.9, and the RBS Measurement section (10 questions) increased from 4.3 to 7.4. These results highlight the intervention’s positive impact on enhancing psychomotor skills across all sections. This enhancement aligns with the objectives of the FAP, which aims to provide experiential learning opportunities and improve community-based healthcare skills among Indian medical graduates [13]. The hands-on nature of the program is a key factor in skill acquisition. Our findings parallel those of Ganapathy et al. [19], who reported that students appreciated the practical exposure and the opportunity to act as healthcare providers. This experience not only enhanced their technical skills but also facilitated meaningful interactions with community members, reinforcing the application of medical knowledge in real-world settings. Furthermore, Ganganahalli et al. [17] found that 86.5% of students believed FAP had provided them with practical skills applicable to medical practice. This finding supports our conclusion that direct engagement in measuring blood pressure, MUAC, infant length and height, weight, and RBS significantly enhanced participants’ psychomotor competencies. Similarly, Shikha et al. [7] highlighted the strengths of FAP in providing hands-on experience, which is crucial for skill retention and proficiency, as demonstrated by the improvement in post-test scores in our study. Reshmi et al. [18] further emphasized that active learning through FAP helps students retain information better by performing the tasks themselves. This perspective aligns with our findings, where participants demonstrated measurable improvements in all assessed psychomotor skills, reinforcing the importance of practical engagement in medical education. Overall, our study corroborates the broader literature on the effectiveness of hands-on, community-based medical education. The observed improvements in psychomotor skills among participants affirm the value of experiential learning, as endorsed by various studies. The integration of such programs into medical curricula can bridge the gap between theoretical knowledge and practical application, ensuring that future healthcare professionals are well-equipped to handle real-world clinical scenarios.

The findings of our study align with existing literature on the FAP, reinforcing its perceived value among medical students. All participants in our study, 150 (100%), found the program useful, a sentiment echoed by Landge et al. [13], where 80% of students considered FAP a valuable activity and expressed willingness to participate throughout their professional years. Similarly, Vairavasolai et al. [14] reported that 99% of students regarded FAP as a positive field experience, underscoring its role in holistic medical education. Rapport with adopted families was largely positive, with 103 (68.7%) participants reporting good rapport and 25 (16.6%) rating it as excellent. However, communication barriers remained a challenge, as also noted by Chhabra et al. [20] and Baruah et al. [16], where students encountered difficulties in gaining trust and cooperation from families. Tripathy et al. [21] similarly emphasized the importance of prolonged interaction with local communities to enhance engagement. In addressing family issues, counselling, 89 (59.3%), and health talks, 72 (48%), emerged as the most commonly employed strategies in our study. These findings align with Ganganahalli et al. [17], where the majority of students recognized FAP’s role in enhancing communication skills and patient-centered care. Additionally, reluctance among certain families to share health information, as observed in our study, is consistent with findings by Reshmi et al. [18], where non-responsiveness was attributed to limited health awareness. The majority of participants, 136 (90.7%), acknowledged FAP’s contribution to enhancing community knowledge, aligning with Vairavasolai et al. [14], where 68% of students believed the program helped shape empathetic and confident physicians. Furthermore, ethical values, accountability, social commitment, and teamwork were highly rated by participants, consistent with the observations of Shikha et al. [7], who identified these attributes as key strengths of FAP. Despite its widely recognized benefits, a small proportion of participants, 3 (2%), perceived FAP as a waste of time, a perspective comparable to the findings of Raja et al. [22], who reported that some students exhibited disinterest in the program.

Furthermore, FAP is expected to develop core professional competencies such as communication skills, empathy, and the ability to deliver healthcare while understanding the customs and culture of the rural population. The program aims to prepare students as primary healthcare consultants and team leaders in healthcare by equipping them with essential skills, including problem diagnosis through hands-on field training, ultimately enhancing their clinical proficiency [18]. Moreover, students strongly believe that the FAP not only facilitates improved healthcare access for rural populations but also cultivates a generation of doctors well aware of the challenges faced by these communities. They have expressed that such an initiative would contribute to better healthcare delivery and enable them to serve as community leaders [14]. Given these benefits, integrating FAP into the MBBS curriculum as a structured component is imperative. This initiative, through village outreach, will be instrumental in shaping “complete doctors” with a humane approach and the confidence to lead socio-medical advancements. The long-term impact of FAP is expected to be visible in the coming years, benefiting both medical education and rural healthcare systems [18].

Limitations

This study has several limitations that should be considered when interpreting the findings. First, it was conducted at a single medical college, which may limit the generalizability of the results to other institutions with different student populations or program implementations. Second, the reliance on self-reported data in the affective domain may introduce response bias, as students might overestimate positive perceptions due to social desirability or institutional expectations. Additionally, the follow-up period was limited to 14 months, which constrains the ability to assess the long-term retention of knowledge and skills acquired through the FAP. The use of purposive sampling could also introduce selection bias, potentially affecting the representativeness of the sample.

## Conclusions

The findings of this study highlight the positive impact of the FAP on the knowledge and psychomotor skills of medical students. The affective domain analysis revealed a strong rapport with adopted families and a high level of perceived usefulness of the program, with students actively engaging in counselling, health education, and motivational activities. FAP was recognized for enhancing community knowledge and providing valuable field exposure, despite some challenges. Ethical values, teamwork, social commitment, and accountability emerged as critical components contributing to the program’s success.

## Appendices

**Table 4:** Study tool.

Knowledge questions			
1.	Family is defined as all of the following except: a) Group of individuals related biologically; b) Group of individuals related by institution of marriage; c) Group of individuals related by blood only; d) Group of individuals living together and eating from the same kitchen	23.	Following are the criteria for sanitary well except: a); Lining; b) Parapet wall; c) Covering; d) No platform
2.	What do you mean by attached house? a) The house which shares one or more walls with another house; b) The house which does not share walls with another house	24.	Sanitation barrier F diagram includes all of the following except: a) Finger; b) Fly; c) Food; d) Air
3.	Ventilation is defined by all of the following except: a) Temperature of incoming air; b) Humidity of incoming air; c) Purity of incoming air; d) Only supply of outdoor air in exchange of vitiated inside air	25.	Sullage is: a) Wood, metal; b) Residue from fire; c) Waste water which does not contain human excreta; d) None of the above
4.	Respiratory diseases can be decreased by using which method for cooking: a) Chulha; b) Gas cylinder; c) Kerosene stove	26.	Sewage is a: a) Wood, metal; b) Waste water containing excreta; c) Waste from kitchen; d) Residue from fire
5.	All are water borne diseases except: a) Typhoid; b) Cholera; c) Dysentery; d) Scabies	27.	Definition of literate is a: a) Person >7 years of age who can read and write in any language; b) Person >7 years of age who can read with understanding in any language; c) Person >7 years of age who can write with understanding in any language; d) Person >7 years of age who can read and write with understanding in any language
6.	Which of the following is not a household water purification method: a) Boiling; b) Chemical disinfection; c) Ozonation; d) Filtration	28.	What do you mean by consanguinity? a) Two persons related by blood; b) Two persons who are from same community
7.	Variables used for calculation of per capita income except: a) Total income of the family; b) Income of head of the family; c) Total number of family members	29.	BP reading of 120–139mmHg systolic and 80–89 mmHg diastolic comes under which category of hypertension classification? a) Normal; b) Prehypertension; c) Hypertension stage 1; d) Hypertension stage 2
8.	Criteria for area to be called as urban area includes: a) Minimum population of 5,000; b) At least 75% of male working population engaged in non-agricultural pursuits; c) Density of population of at least 400 per sq.km; d) All of the above	30.	Cutoff level of hemoglobin to diagnose anemia for pregnant female is: a) 11 g/dL; b) 12 g/dL; c) 11 mg/dL; d) 12 mg/dL
9.	Rural area is defined as: a) Area with density up to 400 per sq. km; b) Minimum of 75% of male working population involved in agriculture activities; c) Panchayat takes all the decisions; d) All of the above	31.	Formula to calculate Body Mass Index is: a) Weight (g)/height (m)^2^; b) Weight (kg)/height (m)^2^; c) Weight (kg)/height (cm)^2^; d) Weight (kg)/height (m)
10.	What is slum? a) All specified areas in a town notified as slum by state/local govt. and UT Administration under any act; b) All areas recognized as slum by state/local govt. and UT Administration, housing and slum boards, which may have not been formally notified as slum under any act; c) Compact area of population of at least 300 or about 60–70 households of poorly built congested tenements, in unhygienic environment usually with inadequate infrastructure and lacking in proper sanitary and drinking water facilities	32	How many households should be preferably adopted by one student as per family adoption programme? a) 3; b) 4; c) 5; d) 2
11.	What is full form of PHC? a) Public health centre; b) Primary healthcare centre	33.	Following are not criteria to decide head of the family except: a) Oldest person; b) Highest earning person; c) Who takes all decisions; d) None of the above
12.	Definition of screening includes all except: a) Search for unrecognized disease; b) By rapidly applied test; c) Among diseased population; d) Among apparently healthy population	34.	Which of the following points express the importance of address of an individual: a) It is used to assess the accessibility to and from the house; b) It is used to identify any health problem that might be associated with the location; c) It is used to assess its distance from important centres such as PDS, Anganwadis, Health centres, etc…; d) All of the above
13.	Which of the following is not included in the types of family: a) Nuclear; b) Joint; c) Three generations; d) Four generations	35.	Arrange the following stages of family cycle in chronological sequence: a) Formation, Extension, Complete extension, Dissolution, Contraction, Complete contraction; b) Formation, Extension, Contraction, Complete extension, Complete contraction, Dissolution; c) Formation, Contraction, Complete contraction, Extension, Complete extension, Dissolution; d) Formation, Extension, Complete extension, Contraction, Complete contraction, Dissolution
14.	Nuclear family consists of: a) Husband, wife and son; b) Husband, wife and dependent children; c) Husband and wife only; d) Father, Mother, Husband and wife	36.	Which of the following are criteria given for overcrowding: a) Floor space area; b) Sex separation; c) Number of rooms; d) All of the above
15.	Which are the different types of houses: a) Pucca house; b) Kutcha house; c) Semi pucca; d) All of the above	37.	The optimum floor space recommended per adult person in a house is a) 50–100 sq.ft.; b) 101–450 sq.ft.; c) 151–200 sq.ft.; d) 201-250 sq.ft.
16.	Which of the following is the correct sequence of various components of the communication process: a) Receiver, Message, Channel, Feedback, Sender; b) Sender, Feedback, Message, Channel, Receiver; c) Sender, Message, Channel, Receiver, Feedback; d) Message, Sender, Channel, Feedback, receiver	38.	Which of the following flies is found in cracks and crevices of walls during daytime a) Sand fly; b) Housefly; c) Blackfly; d) Tse tse fly
17.	Waste water from kitchen is called a) Refuse; b) Garbage; c) Sullage; d) Sewage	39.	Upper class score in Kuppuswamy scale is: a) 5–10; b) 11–15; c) 16–25; d) 26–29
18.	Safe water criteria include all of the following except: a) Free from pathogens; b) Free from harmful chemicals; c) Free from chlorine; d) Free from colour and odour	40.	Body mass index is also known as: a) Quetelet index; b) Broca’s index; c) Ponderal index; d) None of the above
19.	Which of the following flies do not bite a) Sand fly; b) Housefly; c) Blackfly; d) Tse tse fly	41.	Which of the following is not included in vital events: a) Birth; b) Death; c) Accident; d) Divorce
20.	Mosquitoes that breed in dirty water collection are: a) Anopheles; b) Culex; c) Aedes; d) Mansonia	42.	Definition of screening includes all except: a) Search for unrecognized disease; b) By rapidly applied test; c) Among diseased population; d) Among apparently healthy population
21.	Mosquitoes that breed in dirty water collection are: a) Anopheles; b) Culex; c) Aedes; d) Mansonia		
22.	Kuppuswamy scale for socioeconomic status is based on: Income of the family, No. of livestock, No. of acres of farm land Income of the family, No. of members in family, Education of head of family No. of vehicles in family, Occupation of head of family, Education of head of family Income, Occupation of head of family, Education of head of family		
Psychomotor questions			
1.	Which step is correctly sequenced for taking blood pressure? Place the stethoscope over the brachial artery before inflating the cuff. Inflate the cuff to 200 mmHg and then slowly deflate it. Measure blood pressure immediately after the patient has exercised. Position the patient’s arm at heart level with the palm facing down	21.	How should the MUAC measuring tape be tightened during measurement? a) Loose enough to slide easily around the arm; b) Tight enough to indent the skin slightly; c) As tight as possible to get an accurate reading; d) Tightened gradually while measuring
2.	Which action ensures accurate systolic blood pressure measurement? Rapidly inflating the cuff to 180 mmHg. Deflating the cuff at a rate of 2–3 mmHg per second. Using a cuff that is too small for the patient’s arm. Taking the measurement while the patient’s arm is above heart level	22.	What is MUAC measurement range (in cm) for a child to fall within the yellow section, indicating moderate acute malnutrition? a) Less than 11.5 cm; b) 11.5-12.5 cm; c) 12.5-13.5 cm; d) Greater than 13.5 cm
3.	What is the correct procedure for identifying diastolic blood pressure? Listen for the first sound. Note the point where all sounds disappear. Continue deflating until no sound is heard. Record the pressure when the sound is muffled	23.	What is the recommended number of times to measure MUAC for accuracy? Once, to avoid discomfort for the patient. 3 times and averaging the measurements. Twice, and then taking the higher of the two readings. Continuously until consistent measurements are obtained
4.	When measuring blood pressure, the stethoscope should be placed: Over the brachial vein. Directly on the skin above the cuff. On the antecubital fossa. Just below the cuff, over the brachial artery	24.	MUAC measurement is used for age group of: a) 1–5 years; b) 6 months to 5 years; c) 1–5 months; d) 6 months to 2 years
5.	What is the correct placement of the MUAC measuring tape? Just below the elbow joint. Midway-between the shoulder and elbow. Midpoint between the tip of the acromion and olecranon process of ulna. Directly over the bicep muscle	25.	How should the MUAC measurement be recorded? In centimeters, In millimeters, rounding to the nearest centimeter. In inches, converted to centimeters. None of the above
6.	Where should you position the infant for accurate length measurement? Sitting upright on a scale. Lying flat on their back on a measuring board. Standing with support against a wall. Held by the caregiver in a standing position	26.	How should an individual stand while their height is being measured? Facing the stadiometer. Facing away from the stadiometer. With heels, buttocks, shoulders, and head touching the stadiometer. With feet together and hands on hips
7.	Which part of the infant's body should be used to measure length? From the top of the head to the base of the neck. From the top of the head to the heels. From the shoulders to the base of the spine. From the base of the neck to the tip of the toes	27.	To ensure an accurate height measurement, what should the individual do with their head? Tilt it back slightly. Look straight ahead Look down at their feet. Turn it to the side
8.	How should the infant be positioned during length measurement? Arms and legs fully extended. Arms relaxed and legs bent. Arms crossed over the chest. Legs extended, arms bent at the elbows	28.	Which part of the stadiometer should be adjusted to touch the top of the individual’s head? The base plate. The vertical ruler. The horizontal headpiece. The digital display
9.	What is the correct method for recording infant length? Measure to the nearest half inch. Measure in centimeters, rounding to the nearest millimeter. Record the measurement in feet and inches. Estimate the length based on visual assessment	29.	When measuring height, why is it important for the individual to remove footwear and hair style? To reduce weight. To avoid incorrect height measurement. To prevent contamination. For better comfort
10.	Which equipment is used for accurately measuring an individual's height in a clinical setting? Weighing scale Stadiometer Thermometer	30.	How should the stadiometer be positioned relative to the individual for accurate measurement? a) Slightly angled; b) Vertically upright; c) Horizontally flat; d) At 45-degree angle
11.	What is the first step in preparing a stadiometer for height measurement? Asking the individual to remove their shoes. Recording the individual’s weight. Disinfecting the equipment	31.	What should the individual do with their arms while their height is being measured? Keep them at their sides. Cross them over their chest. Raise them above their head. Place them behind their back
12.	What should be not checked before recording the height measurement? The individual’s heart rate. That the headpiece is firmly in place. Calibration on stadiometer. The stadiometer is on hard surface	32.	Which device is used for measuring random blood sugar levels? Sphygmomanometer. Glucometer. Thermometer. Pulse oximeter
13.	Name of the equipment used for measuring an individual’s weight is: Weight meter. Weight stand. Weighing scale	33.	Which is the first step in preparing an individual to measure random blood sugar? Disinfecting the puncture site. Calibrating the glucometer. Selecting the puncture site. Disinfecting the lancet
14.	How should an individual position themselves on a weighing scale? With one foot on the scale. With both feet centered on the scale. With footwear. With heavy weight in hand	34.	What should be done to test strip after display of reading on the glucometer? Wash the strip. Wash the strip and discard. Preserve strip Discard the strip
15.	To ensure an accurate weight measurement, what should the individual remove? Outer clothing and heavy accessories. Glasses and contact lenses Socks. Wristwatch and jewelry	35.	Which part of the finger is recommended for the lancet puncture? The center of the fingertip. The side of the fingertip. The nail bed. The palm
16.	What should be done before asking the individual to step onto the scale? Adjust the scale to zero. Record the individual’s height. Check the room temperature. Ask the individual to hold their breath	36.	What should be done immediately after pricking the finger with the lancet? Squeeze the finger to obtain a blood drop. Clean the puncture site with alcohol. Insert the lancet into the glucometer. Record the blood pressure
17.	How should the weighing scale be positioned for accurate measurement? On a soft surface like carpet. On a hard, level surface. Slightly angled. On sloping surface	37.	How should the blood drop be applied to the test strip? By smearing it across the strip. By allowing the drop to be absorbed at the edge of the strip. By rubbing strip on the puncture site. Placing strip directly into blood drop
18.	What should be done if the weighing scale shows inconsistent readings? Check calibration and replace the batteries. Ignore the readings. Ask the individual to step off and then back on	38.	What should be done if the glucometer displays an error message after applying the blood drop? Ignore the error and record the reading. Discard the test strip and use a new one. Use same strip after wash. Restart the glucometer
19.	How should the puncture site be treated after obtaining the blood sample? Leave it open to air dry. Apply a sterile gauze or bandage. Wash it with soap and water. Apply a cold compress	39.	Why is it important to use a new lancet for each blood sugar measurement? To ensure an accurate reading. To prevent cross-contamination and infection. To reduce pain for the individual
20.	What is the next step after recording the blood sugar reading? Discarding the used lancet in a sharps container. Recalibrating the glucometer. Cleaning the glucometer		
Affective questions			
1.	How is your rapport with the adopted family? a. Poor; b. Good; c. Excellent	15.	How do you support and encourage to the family to solve their problems? a) counselling; b) motivating; c) referral; d) health talk
2.	What is your opinion about family adoption program? a. Useful; b. Not useful	16.	How do you view the process of adopting a family? a. Sifficult and risky process; b. Useful for learning purpose; c. Challenging but rewarding for field exposure; d. Seems too complicated; e. Helpful for rural populations; f. Only considered if absolutely necessary
3.	How do you feel when the family discuss about their own problems? a. Comfortable; b. Not comfortable; c. Eagerness to know; d. Useless	17.	How do you perceive the emotional needs of an adopted family? a. They may have more emotional needs, and I am ready to support them; b. They may have emotional needs, but I’m unsure how to handle them; c. They should adjust according to me; d. I’m concerned their emotional needs could be overwhelming
4.	What do you feel about your adopted family? a. I am concerned about the challenges through which the family is passing; b. I treat them as my own family and fully commit to their growth and happiness; c. I’m not sure how I could be helpful to the family; d. I feel it is useless to work on Family Adoption Program	18.	How comfortably are you discuss your views with family? a. I openly discuss my views; b. I discuss it if necessary; c. I avoid discussing; d. I’m not sure how I feel about discussing it
5.	How you respond to the family if they do not cooperate to give you proper answers? a) take it as a challenge; b) try to convince; c) leave that family	19.	How do you view the potential challenges of adoption? a. Prepared to face challenges with patience; b. Challenges worry me, but can be managed; c. Challenges might overwhelm me; d. I can’t handle the challenges of FAP
6.	Which statement best reflects your attitude towards family adoption programme? a. Adoption enhances community knowledge; b. It’s a waste of time; c. Uncertain of its impact on my study	20.	How do you show respect towards family adopted under family adoption program? a) Listening actively; b) Enforcing strict rules; c) Providing frequent updates; d) Offering diverse options
7.	How emotionally ready do you feel to adopt a family? a. I feel fully prepared and excited about the family adoption programme; b. I think I’m ready, but I have some concerns; c. I’m not sure if I’m emotionally prepared for adoption; d. I don’t think I’m emotionally ready for such a responsibility	21.	Do you actively listen to the concerns and needs of families in the program? a. Yes; b. No
8.	How do you maintain professional boundaries while building relationships with adopted families? a) Maintaining confidentiality; b) Keeping friendly attitude; c) Following up regularly; d) Encouraging informal communication	22.	Which of the following behaviors best exemplifies leadership in a family adoption program? a) Providing clear guidance; b) Encouraging team collaboration; c) Making informed decisions; d) Setting high standards
9.	Which of the following do you consider that it is best consistent communication (reliability) with the families involved in the family adoption program? a) Promptly returning calls; b) Offering flexibility; c) Providing detailed information; d) Consistently following procedures	23.	Strong ethical values are how important in shaping your approach to a family adoption program? Most important; Moderately important; Not important
10.	Do you consistently communicate with the adopted families? a. Yes; b. No	24.	In the context of a family adoption program, which of the following best reflects a patient-centered attitude? Standardizing the adoption process without considering individual family situations. Focusing on the needs and preferences of the families involved in the program. Prioritizing the legal and financial aspects of adoption over the emotional needs of the family. All of the above
11.	Which of the following best demonstrates effective communication in the context of a family adoption program? Using technical jargon when discussing with families. Providing families with clear, empathetic, and timely information about the adoption program. Limiting communication to only necessary legal updates, avoiding personal discussions. Only communicating with families when there is a major issue in the family adoption program	25.	How important is accountability in the success of a family adoption program? a. Most important; b. Moderately important; c. Not important
12.	Do you believe that being a part of family adoption program has contributed for your professional growth? a. Yes; b. No	26.	How responsiveness is significant in ensuring the success of a family adoption program? a. Most important; b. Moderately important; c. Not important
13.	Should the family adoption program target the improvement in social behaviour of all family members? a. Yes; b. No	27.	How does social commitment influence your attitude towards family adoption programs? a. Most important; b. Moderately important; c. Not important
14.	How do you view the role of teamwork in a family adoption program? Most important; Moderately important; Not important	28.	How do you perceive the responsibility associated with participation in a family adoption program? a. Most important; b. Moderately important; c. Not important
